# Supplementary material for: Hypercaloric diet models do not develop heart failure, but the excess sucrose promotes contractility dysfunction
Source: PLoS One. 2020 Feb 7;15(2):e0228860. doi: 10.1371/journal.pone.0228860 (PMC7006916; doi:10.1371/journal.pone.0228860)
Supplement: Attachment — Submitted filename: Response to Reviewers.doc [file pone.0228860.s002.doc]

**Manuscript Number:** PONE-D-19-28274R1

**Title:** Hypercaloric diet models do not develop heart failure, but the excess sucrose promotes contractility dysfunction

**Review Comments to the Author**

**Reviewer #2:**

**The authors have reviewed the manuscript entitled “Hypercaloric diet models do not develop heart failure, but the excess sucrose promotes contractility dysfunction” that aimed to develop a hypercaloric animal model of cardiac remodeling and predisposition to heart failure. The authors answered to some of the questions raised, however they failed to include the requested data into the manuscript and to add results that will give information on the mechanisms that may explain the differences between high fat and high sucrose diets.**

**Please see below my comments:**

**1 - Although some of the information requested by this reviewer was already included in a previous publication as stated by the authors “Matias AM, Estevam WM, Coelho PM, Haese D, Kobi JBBS, Lima-Leopoldo AP, Leopoldo AS. Differential Effects of High Sugar, High Lard or a Combination of Both on Nutritional, Hormonal and Cardiovascular Metabolic Profiles of Rodents. Nutrients. 2018 Aug 11;10(8). pii: E1071. doi: 10.3390/nu10081071.” e.g. composition of the diets, caloric intake, diet administration, this must be included in the present manuscript to allow the readers a full understand of the manuscript. This information is crucial to the manuscript as it main focus is to evaluate the effect of different diets on heart failure and cardiac contractility.**

Dear Reviewer, we appreciate the comment and as requested the composition of the diets, caloric intake and diet administration were added in the text. In addition, the Table 1 was added in the current manuscript.

The added sentence about the composition of the diets was *“**The experimental diets provided sufficient amounts of protein, vitamins and minerals according to the Nutrient Requirements for Laboratory Animals. The diets used in the current study were formulated by Nutriave Alimentos® (Vitória, Espírito Santo, Brazil). The feed ingredients were blended, homogenized and extruded (Extru-Tech Extruder, Model E-750, Sabetha, KS, USA) in the form of pellets. Then, the pellets were dried on a horizontal conveyor dryer (20 minutes, temperature: ±70° C). The composition (g/kg) and nutrients for each experimental diet (%) are described in Table 1.”* **(Material and Methods, Animal care and Experimental design, page 5, line 3-9)**

**Table 1.** Composition and nutritional values of diets

| **Components (g/kg)** | **Diets** | | | |
| --- | --- | --- | --- | --- |
|  | **C** | **HS** | **HF** | **HFHS** |
| **Corn** | 200 | 200 | 180 | 80 |
| **Rice** | 200 | 200 | 200 | 200 |
| **Bone meal** | 120 | 120 | 120 | 120 |
| **Sugar** | - | 100 | - | 100 |
| **Soy oil** | 75 | 75 | - | - |
| **Lard** | - | - | 200 | 200 |
| **Gluten** | 200 | 200 | 200 | 200 |
| **Salt** | 3.5 | 3.5 | 3.5 | 3.5 |
| **Mineral Mix**** | 35 | 35 | 30 | 30 |
| **Vitamin Mix**** | 16.5 | 16.5 | 16.5 | 16.5 |
| **Inert Material***** | 150 | 50 | 50 | 50 |
| **Total (g)** | **1000** | **1000** | **1000** | **1000** |
| **Nutrient Composition (%)** |  |  |  |  |
| **Protein** | 24.8 | 21.8 | 17.8 | 19.2 |
| **Carbohydrate** | 49.6 | 52.3 | 44.6 | 43.4 |
| **Lipids** | 25.6 | 25.9 | 37.6 | 37.4 |
| **Energy Density (Kcal/g)** | **3.55** | **3.65** | **4.59** | **4.49** |

Diets. C: normal rodent chow; HS: High-sugar; HF: high-fat; HFHS: high fat and high sugar. *Rats received diet with simple carbohydrate and water supplemented with sugar (300 g/L) in alternate weeks. In order to calculate the caloric intake of HS, the caloric value of the sugar diet (3.65 kcal/g) plus the caloric value of water intake with sugar (1.2 kcal/ml).

As requested by Reviewer, in order to analyze if dietary-induced obesity was associated with alterations in the nutritional behavior, the food consumption (FC) was measured daily and the calorie intake (CI) was calculated weekly by the average weekly FC × dietary energetic density. These information and description of results were added in the Table 2 and results section. **(Results, page 9, line 1-9)**

The added sentence about the nutritional behavior was “*The nutritional profile of rats is summarized in Table 2. The C rats had an approximately 17.9%, 29.6% and 37.6% greater daily food consumption (g) than the HF, HFHS and HS groups, respectively, but the daily caloric intake was higher in the HS group in relation to the C and HFHS groups (HS: 92.1 ± 2.1 vs. C: 79.2 ± 2.6 and HFHS: 77.3 ± 1.8 kcal/day, p < 0.05). In addition, there was a difference in FC between rats fed the HF diet compared with the HS group since rats fed the HS diet consumed significantly less food (HG: 16.2 ± 0.5 vs. HF:18.9 ± 0.7; p < 0.05). Furthermore, HF presented a 12.4% increase in caloric intake over HFHS (p < 0.05). There was no difference in the caloric intake of C compared to HF and HFHS (p > 0.05).”*

**Table 2.** General Characteristics

| **Variables** | **Experimental Groups** | | | |
| --- | --- | --- | --- | --- |
|  | **C** | **HS** | **HF** | **HFHS** |
| **IBW (g)** | 107 ± 3 | 110 ± 3 | 111 ± 3 | 110 ± 4 |
| **FBW (g)** | 533 ± 17 | 538 ± 13^#^ | 649 ± 34* | 616 ± 22 |
| **BW gain (g)** | 426 ± 17 | 428 ± 12^#^ | 538 ± 32* | 506 ± 20 |
| **Epididymal fat pad (g)** | 11.2 ± 0.6 | 10.5 ± 0.7 | 13.1 ± 0.9 | 13.5 ± 1.0 |
| **Visceral fat pad (g)** | 11.4 ± 0.6 | 10.6 ± 0.6^#&^ | 18.5 ± 1.4* | 15.9 ± 1.4* |
| **Retroperitoneal fat pad (g)** | 21.9 ± 1.0 | 21.8 ± 2.0^#&^ | 40.4 ± 4.5* | 34.4 ± 2.2* |
| **Body fat (g)** | 44.5 ± 1.6 | 42.9 ± 0.6^#&^ | 72.0 ± 6.3* | 63.8 ± 4.3* |
| **Adiposity index (%)** | 8.3 ± 0.2 | 7.9 ± 0.3^#&^ | 10.9 ± 0.5* | 10.2 ± 0.3* |
| **Food consumption (g/day)** | 22.3 ± 0.7 | 16.2 ± 0.5 | 18.9 ± 0.7 | 17.2 ± 0.4 |
| **Caloric intake (kcal/day)** | 79.2 ± 2.6 | 92.1 ± 2.1 | 86.9 ± 3.5 | 77.3 ± 1.8 |
| **Glucose (mg/dL)** | 108 ± 2 | 112 ± 3 | 115 ± 4 | 115 ± 3 |
| **Insulin (ng/mL)** | 1.86 ± 0.13 | 1.77 ± 0.15 | 2.19 ± 0.16 | 2.39 ± 0.23 |

Data are presented as the mean ± SEM. Control diet - (C; n=12); high-sugar diet - (HS; n=14); high-fat diet - (HF; n=13), and high-fat and high-sugar diet (HFHS; n=13). IBW: initial body weight; FBW: final body weight; BW: body weight. One-way ANOVA for independent samples followed by Tukey *post hoc* test. p < 0.05 *vs.* * C; ^#^ HF *vs.* HS; ^&^ HFHS *vs.* HS.

Dear Reviewer, we appreciate the commentary and as requested, the information about how was the HFHsu administered was mentioned below and added in the text.

*All animals had free access to water and chow (40 g/day). To analyze whether dietary-induced obesity was associated with alterations in nutritional behavior, food consumption (FC) was measured daily. Calorie intake (CI) was calculated weekly by the average weekly FC × dietary energetic density. The HS group had water supplemented with sugar (300 g/l) in alternate weeks. For the calculation of the caloric intake of the HS group, the caloric energy from the water supplemented with sugar was also quantified (1.2 kcal/mL consumed).* **(Material and Methods, Animal care and Experimental design, page 4, line 2-7)**

**2 - Please include data of metabolic profile of the different diets, mainly insulin and glycemia values, specially in the context of the discussion of the mechanisms promoting cardiac remodeling (lines 263- 282 page 12)**

Dear Reviewer, we appreciate the commentary and as requested, we included data of metabolic profile (insulin and glucose) of the different diets. This information was added in the Table 2, results and discussion sections. The sentence added was described below.

*The metabolic profile parameters including glucose (p=0.26) and insulin (p=0.06) were similar among the groups (Table 2).* **(Results section, page 9, lines 9-10)**

**Table 2.** General Characteristics

| **Variables** | **Experimental Groups** | | | |
| --- | --- | --- | --- | --- |
|  | **C** | **HS** | **HF** | **HFHS** |
| **IBW (g)** | 107 ± 3 | 110 ± 3 | 111 ± 3 | 110 ± 4 |
| **FBW (g)** | 533 ± 17 | 538 ± 13^#^ | 649 ± 34* | 616 ± 22 |
| **BW gain (g)** | 426 ± 17 | 428 ± 12^#^ | 538 ± 32* | 506 ± 20 |
| **Epididymal fat pad (g)** | 11.2 ± 0.6 | 10.5 ± 0.7 | 13.1 ± 0.9 | 13.5 ± 1.0 |
| **Visceral fat pad (g)** | 11.4 ± 0.6 | 10.6 ± 0.6^#&^ | 18.5 ± 1.4* | 15.9 ± 1.4* |
| **Retroperitoneal fat pad (g)** | 21.9 ± 1.0 | 21.8 ± 2.0^#&^ | 40.4 ± 4.5* | 34.4 ± 2.2* |
| **Body fat (g)** | 44.5 ± 1.6 | 42.9 ± 0.6^#&^ | 72.0 ± 6.3* | 63.8 ± 4.3* |
| **Adiposity index (%)** | 8.3 ± 0.2 | 7.9 ± 0.3^#&^ | 10.9 ± 0.5* | 10.2 ± 0.3* |
| **Food consumption (g/day)** | 22.3 ± 0.7 | 16.2 ± 0.5 | 18.9 ± 0.7 | 17.2 ± 0.4 |
| **Caloric intake (kcal/day)** | 79.2 ± 2.6 | 92.1 ± 2.1 | 86.9 ± 3.5 | 77.3 ± 1.8 |
| **Glucose (mg/dL)** | 108 ± 2 | 112 ± 3 | 115 ± 4 | 115 ± 3 |
| **Insulin (ng/mL)** | 1.86 ± 0.13 | 1.77 ± 0.15 | 2.19 ± 0.16 | 2.39 ± 0.23 |

Data are presented as the mean ± SEM. Control diet - (C; n=12); high-sugar diet - (HS; n=14); high-fat diet - (HF; n=13), and high-fat and high-sugar diet (HFHS; n=13). IBW: initial body weight; FBW: final body weight; BW: body weight. One-way ANOVA for independent samples followed by Tukey *post hoc* test. p < 0.05 *vs.* * C; ^#^ HF *vs.* HS; ^&^ HFHS *vs.* HS.

*Specifically, the HS did not promote cardiac remodeling, however, it was able to prevent the elevation on heart and LV weights in the HFHS, interfering in the effect of HF diet. According to literature, high sucrose intake [68% total energy sucrose diet, 69% total mass sucrose diet, or 20% sucrose solution] induced hyperinsulinemia without alterations in plasma glucose level in rats and mice [28-30]. Interestingly, our HS rats have not demonstrated alterations in glucose and insulin levels (Table 2). Several mechanisms have been postulated, including the prohypertrophic effects of insulin, insulin growth factor-1, and insulin resistance [31]. Initially the circulating insulin levels are increased, directly stimulating cardiomyocyte growth [32] and indirectly via binding to the insulin growth factor-1 receptor [33]. Chess et al. [26], analyzing the effects of dietary extremes (high carbohydrate and fat intake) on the remodeling process and heart failure, reported that high sugar intake causes hyperglycemia in the bloodstream and cardiotoxic effects. Increased glycemia leads to elevated serum insulin levels, which in cardiac tissue, induces increased protein synthesis of cardiomyocytes and, consequently, left ventricular hypertrophy. Sharma et al. [9] propose that carbohydrate intake, in particular sugar, associated or not with pressure overload may cause LV hypertrophy, via insulin receptor stimulation and activation of Akt/mTOR, proteins involved in protein signaling pathways. Thus, our results from HS diet suggest that the sugar intake was not able to affect the process of cardiac remodeling, probably due to the absence of hyperinsulinemia and elevation of glucose levels, as well as it was not promote cardiotoxic effect, indicating a cardioprotective effect alone or when associated with the HF diet.* **(Discussion section, page 13, line 12)**

**3 - Please include descriptions for figure legends. e.g – Figure 1 – effect of different diet composition on…**

Dear Reviewer, we appreciate the commentary and as requested, we included the descriptions for all figures (Figs. 1-4).

The description of figures is described below, and it was added in the text:

**Figure 1.** Effect of different diet composition on cardiac remodeling. Data are shown as mean ± SEM. Control diet - (C; n=5); high-sugar diet - (HS; n=8); high-fat diet - (HF; n=5), and high-fat and high-sugar diet (HFHS; n=6). HW: heart weight; LW: left weight. p < 0.05 *vs.* ^#^ HF *vs.* HS. One-way analysis of variance (ANOVA) followed by the Tukey *post hoc* test.

**Figure 2.** Histological study in myocardium from control diet - (C; n=5); high-sugar diet - (HS; n=8); high-fat diet - (HF; n=5), and high-fat and high-sugar diet (HFHS; n=6) after 20 weeks. (A) cross sectional area (CSA). (B): interstitial collagen of myocardium; representative *picrosirius red-stained* left ventricle (LV) section. Arrows: interstitial collagen. Data are shown as mean ± SEM. One-way analysis of variance (ANOVA) followed by the Tukey *post hoc* test. p < 0.05 *vs.* * C; ^&^ HFHS *vs.* HS; ^§^HFHS *vs.* HF.

**Figure 3.** Effect of different diet composition on parameters of heart failure. Data are shown as mean ± SEM. Control diet - (C; n=12); high-sugar diet - (HS; n=14); high-fat diet - (HF; n=13), and high-fat and high-sugar diet (HFHS; n=13). RV: right ventricle. (A) Lung weight/body weight ratio. (B) Right lung weight/body weight ratio. (C) RV weight/body weight. (D) Lung water content (C = 5; HS = 8; HF = 5, and HFHS = 6). One-way analysis of variance (ANOVA) followed by the Tukey *post hoc* test.

**Figure 4.** Effect of different diet composition on contractile function and calcium transients of left ventricular cardiomyocytes from control diet (C; n= 5; cells= 71), high-sugar diet (HS; n=6, cells= 115), high-fat diet (HF; n= 6, cells= 106) and high-fat and high-sugar diet (HFHS; n=5, cells= 81). Data are shown as mean ± SEM. (A) Representative contraction traces obtained from the cardiomyocytes of rats: (B) Cell shortening expressed as % of resting cell length. (C) Time to 50% of contraction. (D) Time to 50% of relaxation. (E) Amplitude of transients. (F) Time to peak. (G) Time to from peak transient to half resting value p < 0.05 *vs.* * C; ^#^ HS *vs.* HF; ^&^ HFHS *vs.* HS. One-way analysis of variance (ANOVA) followed by the Tukey *post hoc* test.
