# Supplementary material for: Hypercaloric diet models do not develop heart failure, but the excess sucrose promotes contractility dysfunction
Source: PLoS One. 2020 Feb 7;15(2):e0228860. doi: 10.1371/journal.pone.0228860 (PMC7006916; doi:10.1371/journal.pone.0228860)
Supplement: Attachment — Submitted filename: Response to Reviewers.doc [file pone.0228860.s003.doc]

**Manuscript Number:** PONE-D-19-28274R2

**Title:** Hypercaloric diet models do not develop heart failure, but the excess sucrose promotes contractility dysfunction

**Review Comments to the Author**

**Reviewer #2: The authors have reviewed the manuscript entitled “Hypercaloric diet models do not develop heart failure, but the excess sucrose promotes contractility dysfunction” that aimed to develop a hypercaloric animal model of cardiac remodeling and predisposition to heart failure. The authors have answered adequately to some of the questions raised however I have 2 additional comments:**

**1- I really don’t understand how the animals HFHS that have a decreased caloric intake in comparison with the controls gain weight and have an increased body fat and adiposity index compared with controls. (table 2)**

Dear Reviewer, we appreciate the commentary and as requested, the explanation about how the animals HFHS have a decreased caloric intake, but higher body fat and adiposity index is related to feed efficiency, which it is the ability to transform consumed calories into body weight, was determined by following the formula: mean body weight gain (g)/total calorie intake (kcal). In this sense, the feed efficiency (%) was higher in the HF (14.3%) and HFHS groups (20.8%) than in C (*data included in the Table 2*) **as demonstrated (HFHS: 4.64 ± 0.09 and HF: 4.39 ± 0.09 vs. C: 3.84 ± 0.08; p < 0.05).** The elevation in body weight and adiposity in the hyperlipidic experimental models (HF) increased with sugar, refers to caloric intake and food efficiency which, generally, lead to a greater availability of calories and hyperphagia [1,2]. Even without an increase in caloric intake, it is possible to develop obesity, since the change in nutrient composition influences the efficiency of food utilization, thus leading to an increase in fat storage per calorie consumed. The elevation in body fat may be due to an increase in the caloric density of the diet, which may lead to a higher total caloric intake or an increase in the intake of a given macronutrient [3]. Although they had a similar total caloric intake, the HF and HFHS groups consumed 61% and 43% more calories from fat, respectively, than the C animals did. Therefore, the elevation of feed efficiency in HFHS could explain how the animals HFHS had an increased body fat and adiposity index compared with C rats, despite the absence of alterations in caloric intake among HFHS and HF in relation to C group (Table 2).

1. Dourmashkin J, Chang G, Gayles E, Hill J, Fried S, Julien C, Leibowitz S. Different forms of obesity as a function of diet composition. Int. J. Obes. 2005;29:1368–1378.
2. White S, Cercato LM, Araujo D, Souza LA, Soares AF, Barbosa APO, et al. Modelo de obesidade induzida por dieta hiperlipidica e associada à resistência à ação da insulina e intolerância a glicose. Arq Bras Endocrinol Metab 2013;57:339–345.
3. Pereira-Lancha LO, Campos-Ferraz PL, Lancha AH. Obesity: Considerations about etiology, metabolism, and the use of experimental models. Diabetes Metab Syndr Obes. 2012;5:75–87.

The added sentences and Table 2 were detailed below:

In order to analyze if dietary-induced obesity was associated with alterations in the nutritional behavior, the food consumption (FC) was measured daily and the calorie intake (CI) was calculated weekly by the average weekly FC × dietary energetic density, as well as the feed efficiency to show the higher body fat and adiposity index in HF and HFHS in comparison to C group (Table 2). These information and description of results were added in the Table 2 and results section. **(Results, page 9, lines 1-11)**

The added sentence about the nutritional behavior was “*The nutritional profile of rats is summarized in Table 2. The C rats had an approximately 17.9%, 29.6% and 37.6% greater daily food consumption (g) than the HF, HFHS and HS groups, respectively, but the daily caloric intake was higher in the HS group in relation to the C and HFHS groups (HS: 92.1 ± 2.1 vs. C: 79.2 ± 2.6 and HFHS: 77.3 ± 1.8 kcal/day, p < 0.05). In addition, there was a difference in FC between rats fed the HF diet compared with the HS group since rats fed the HS diet consumed significantly less food (HG: 16.2 ± 0.5 vs. HF:18.9 ± 0.7; p < 0.05). Furthermore, HF presented a 12.4% increase in caloric intake over HFHS (p < 0.05). There was no difference in the caloric intake of C compared to HF and HFHS (p > 0.05). While the feed efficiency (%) was higher in the HF (14.3%) and HFHS groups (20.8%) than in C (Table 2), there was a lower feed efficiency in HS rats than C (HS: 3.32 ± 0.05 vs. C: 3.84 ± 0.08; p < 0.05).”* **(Results, pages 9 and 10, lines 1-11)**

**Table 2.** General Characteristics

| **Variables** | **Experimental Groups** | | | |
| --- | --- | --- | --- | --- |
|  | **C** | **HS** | **HF** | **HFHS** |
| **IBW (g)** | 107 ± 3 | 110 ± 3 | 111 ± 3 | 110 ± 4 |
| **FBW (g)** | 533 ± 17 | 538 ± 13^#^ | 649 ± 34* | 616 ± 22 |
| **BW gain (g)** | 426 ± 17 | 428 ± 12^#^ | 538 ± 32* | 506 ± 20 |
| **Epididymal fat pad (g)** | 11.2 ± 0.6 | 10.5 ± 0.7 | 13.1 ± 0.9 | 13.5 ± 1.0 |
| **Visceral fat pad (g)** | 11.4 ± 0.6 | 10.6 ± 0.6^#&^ | 18.5 ± 1.4* | 15.9 ± 1.4* |
| **Retroperitoneal fat pad (g)** | 21.9 ± 1.0 | 21.8 ± 2.0^#&^ | 40.4 ± 4.5* | 34.4 ± 2.2* |
| **Body fat (g)** | 44.5 ± 1.6 | 42.9 ± 0.6^#&^ | 72.0 ± 6.3* | 63.8 ± 4.3* |
| **Adiposity index (%)** | 8.3 ± 0.2 | 7.9 ± 0.3^#&^ | 10.9 ± 0.5* | 10.2 ± 0.3* |
| **Food consumption (g/day)** | 22.3 ± 0.7 | 16.2 ± 0.5*^#^ | 18.9 ± 0.7* | 17.2 ± 0.4* |
| **Caloric intake (kcal/day)** | 79.2 ± 2.6 | 92.1 ± 2.1*^&^ | 86.9 ± 3.5 | 77.3 ± 1.8^α^ |
| **Feed efficiency (%)** | 3.84 ± 0.08 | 3.32 ± 0.05* | 4.39 ± 0.09*^#^ | 4.64 ± 0.09*^&^ |
| **Glucose (mg/dL)** | 108 ± 2 | 112 ± 3 | 115 ± 4 | 115 ± 3 |
| **Insulin (ng/mL)** | 1.86 ± 0.13 | 1.77 ± 0.15 | 2.19 ± 0.16 | 2.39 ± 0.23 |

Data are presented as the mean ± SEM. Control diet - (C; n=12); high-sugar diet - (HS; n=14); high-fat diet - (HF; n=13), and high-fat and high-sugar diet (HFHS; n=13). IBW: initial body weight; FBW: final body weight; BW: body weight. One-way ANOVA for independent samples followed by Tukey *post hoc* test. p < 0.05 *vs.* * C; ^#^ HF *vs.* HS; ^&^ HFHS *vs.* HS, ^α^ HF *vs.* HFHS.

Dear Reviewer, the information about how was the analyze of feed efficiency is mentioned below and added in the text.

*All animals had free access to water and chow (40 g/day). To analyze whether dietary-induced obesity was associated with alterations in nutritional behavior, food consumption (FC) was measured daily. Calorie intake (CI) was calculated weekly by the average weekly FC × dietary energetic density. Feed efficiency (FE), the ability to transform consumed calories into body weight, was determined by following the formula: mean body weight gain (g)/total calorie intake (kcal). The HS group had water supplemented with sugar (300 g/l) in alternate weeks. For the calculation of the caloric intake of the HS group, the caloric energy from the water supplemented with sugar was also quantified (1.2 kcal/mL consumed).* **(Material and Methods, Animal care and Experimental design, page 4, line 2-10)**

**2- Please include in methods section, the methods to analyze insulin and glucose.**

Dear Reviewer, we appreciate the comment and as requested the methods to analyze insulin and glucose was added in the methods section.

The added sentence is described below:

*Metabolic and Hormonal Measurements*

*After 20 weeks, the animals were subjected to 12–15 h of fasting, and blood samples were collected in dry tubes. The serum was separated by centrifugation at 10,000 rpm for 10 min. (Heraeus Megafuge 16R Centrifuge, Thermo Scientific, Massachusetts, USA) and stored at −80 °C for subsequent analysis (Coldlab Ultra Freezer CL374-86V, Piracicaba, São Paulo, Brazil). Serum glucose concentration was measured using specific kit (Bioclin Bioquímica®, Belo Horizonte, Minas Gerais, Brazil and Synermed do Brasil Ltda., São Paulo, Brazil) and analyzed by automated biochemical equipment BS-200 (Mindray do Brasil-Comércio and Distribuição de Equipamentos Médicos Ltda., São Paulo, Brazil). Insulin was determined using an enzyme-linked immunosorbent assay (ELISA) using specific kit (Linco Research Inc., St. Louis, MO, USA). The reading was carried out using a microplate reader (Asys Expert Plus Microplate Reader, Cambourne, Cambridge, UK).* **(Material and Methods, Metabolic and Hormonal Measurements, page 6, lines 1-11)**
